# Social determinants of health and progression to cardio–renal–metabolic multimorbidity and mortality in people with prediabetes: A prospective cohort study

**DOI:** 10.1111/dom.70067

**Published:** 2025-08-27

**Authors:** Rong Hua, Aimin Yang, Le Gao, Elaine Chow, Wenjing Ji, Yin Ting Cheung

**Affiliations:** ^1^ School of Pharmacy, Faculty of Medicine The Chinese University of Hong Kong Hong Kong SAR China; ^2^ Department of Medicine and Therapeutics The Chinese University of Hong Kong, Prince of Wales Hospital Hong Kong SAR China; ^3^ Hong Kong Institute of Diabetes and Obesity The Chinese University of Hong Kong, Prince of Wales Hospital Hong Kong SAR China; ^4^ Department of Pharmacy Administration and Clinical Pharmacy, School of Pharmacy Xi'an Jiaotong University Xi'an China; ^5^ Phase 1 Clinical Trial Centre The Chinese University of Hong Kong, Prince of Wales Hospital Hong Kong SAR China

**Keywords:** cardiovascular disease, cohort study, diabetes complications, type 2 diabetes

## Abstract

**Aims:**

Social determinants of health (SDHs) have been increasingly recognised as upstream drivers of preventable health disparities. However, it is unclear what the role of SDHs is in the whole progression of prediabetes. We aimed to delineate the impact of SDHs on the progression from prediabetes to subsequent cardio–renal–metabolic disease (CRMD), cardio–renal–metabolic multimorbidity (CRMM) and mortality.

**Materials and Methods:**

37 098 participants with prediabetes and free of any CRMD (i.e., type 2 diabetes, cardiovascular disease and chronic kidney disease [CKD]) at baseline from the UK Biobank were included. A combined SDH score was assessed by a sum of 17 items including financial, education, healthcare, neighbourhood, and social domains, and was categorised into favourable, medium, and unfavourable groups by tertile. Outcomes included first CRMD, CRMM (the coexistence of at least two CRMDs) and death during follow‐up. Five transition paths were considered, and multistate models were performed.

**Results:**

Compared with the favourable SDH group, the unfavourable SDH group consistently showed elevated risks for different transition stages, except for the CRMM‐to‐death transition. Lifestyle partially mediated the associations, while the mediation proportions at each stage explained less than 20%. Among disease‐specific associations, unfavourable SDHs disproportionally increased the risks of three CRMDs, with the highest risk observed for the CKD‐to‐death transition (hazard ratio = 2.22, 95% confidence interval: 1.16–4.26).

**Conclusions:**

Unfavourable SDHs were associated with increased risks of progression from prediabetes. Resource allocation and lifestyle promotion should be prioritised for those with unfavourable SDHs to mitigate disparities in progression to CRMM and death in diabetes care, especially in the early disease stage.

## 1 INTRODUCTION

Prediabetes is an intermediate state between normoglycaemia and diabetes. Globally, up to 464 million people were affected by prediabetes in 2021, and this number is estimated to rise to 638 million by 2045.^1^ Studies have shown that prediabetes directly increased the risks of cardiovascular disease (CVD) and chronic kidney disease (CKD),^2^, ^3^ whereas progression to diabetes explained only less than one quarter of the risks of these clinical outcomes.^2^ Therefore, routine screening and early intervention in the prediabetes stage are essential to prevent or delay the progression of diseases such as CVD, type 2 diabetes (T2D), and CKD, which share similar metabolic risk factors and pathophysiology profiles.^4^

Over the past three decades, the global number of people with CVD has substantially increased from 271 million in 1990 to 612 million in 2021.^5^ Concurrently, the number of people with diabetes and CKD has also surged dramatically, reaching 526 and 674 million, respectively.^5^ With a growing aging population, people are facing an increasing risk of first cardio–renal–metabolic disease (FCRMD) progressing to cardio–renal–metabolic multimorbidity (CRMM). Nearly 25% of US older adults were affected by CRMM, and the CRMM contributed to the cumulative risk of disability and mortality.^4^, ^6^, ^7^ Therefore, attention has been shifted to early CRMM prevention and care in a holistic and life‐course approach. Prediabetes offers a critical time window for intervention to prevent progression to diabetes and subsequent CRMM.^4^, ^8^ Although intensive lifestyle modifications and/or pharmacotherapy can help prevent or delay incident diabetes, their preventive effects against major clinical events and mortality have been inconclusive.^9^, ^10^ Personalised factors that may influence the effects of preventive strategies should be considered to provide tailored recommendations and interventions.

The American Heart Association (AHA) has advocated an integrated and interdisciplinary care framework to address CRMM, with social determinants of health (SDHs) screening as a critical component for facilitating risk stratification and reducing care fragmentation.^4^, ^8^ Emerging evidence supports the critical role of SDHs as upstream drivers of preventable health disparities. SDHs are “*conditions in which people grow, live, and work that are shaped by the distribution of money, power, and resources*.^11^” Their influence on downstream health outcomes may outweigh those of lifestyle and medical care.^12^ Previous studies have demonstrated the detrimental impact of specific SDH on diabetes‐related outcomes.^11^, ^13^, ^14^ For example, low educational attainment and unfavourable economic status were associated with a higher incidence of T2D and T2D‐related complications.^13^ It is worthwhile to note that disadvantaged SDHs often coexist and act in complex and intercorrelated ways; for example, glucose control could be worse due to housing insecurity, but may also be exacerbated by food scarcity, inadequate social networks, poor health literacy, and other social risk factors that are interlinked.^15^ Consequently, precisely quantifying the contribution of individual SDH is difficult and rarely useful in identifying the most vulnerable groups.

To capture the synergistic effects of social risk factors, studies have developed a polysocial risk score integrating multifaceted information that more accurately reflects an individual's overall SDH level.^15^ A United Kingdom (UK) Biobank‐based study constructed a combined SDH score and demonstrated its associations with increased risks of vascular complications and mortality in people with T2D.^16^ The effect of this score on progression from prediabetes to CRMM remains unclear, as studies have predominantly investigated outcomes separately (e.g., incidence of specific FCRMD) or only focused on one transition stage (e.g., from T2D to CVD or mortality).^12^, ^16^, ^17^, ^18^ As the CRMD burden accumulates over time, understanding the associations between SDHs and the dynamics of progression to subsequent CRMM and death is crucial to guide the development of targeted primary and secondary prevention and personalised care at each disease stage.

Leveraging a large population‐based cohort, we aimed to delineate the effect of SDHs on progression from prediabetes to subsequent CRMD, CRMM, and mortality in a real‐world setting.

## 2 METHODS

### 2.1 Study population

The UK Biobank is a prospective cohort study that enrolled over 500 000 participants aged 40–69 across the UK between 2006 and 2010. It received approval from the North West Multi‐Centre Research Ethics Committee (reference number: 21/NW/0157), and all participants provided written informed consent. Details regarding its conception and methodologies were documented elsewhere.^19^ The current analysis was approved by the Survey and Behavioural Research Ethics Committee of the Chinese University of Hong Kong (Reference no. SBRE‐24‐0545), and was reported according to the STrengthening the Reporting of OBservational studies in Epidemiology (STROBE) guidelines.^20^

We used the glycated haemoglobin (HbA1c) concentration (5.7%–6.4%, equivalent to ≥39 to <48 mmol/mol) to define prediabetes^21^ as the blood samples were mainly collected under non‐fasting conditions in the UK Biobank. As shown in the flowchart (Figure S1), among 68 378 (13.6%) participants identified with prediabetes at baseline, 13 279 were excluded as they were diagnosed with CRMD before/at baseline, and 4185 were excluded as they had an estimated glomerular filtration rate (eGFR) <60 mL/min/1.73 m^2^ or urinary albumin to urinary creatinine ratio ≥30 mg/g at baseline, indicative of CKD.^22^ We further excluded those who had missing data for any component of SDHs (*n* = 13 117) and lifestyle (*n* = 699). Finally, 37 098 participants were included in this study. Table S1 presents the numbers of people with missing data for each SDH/lifestyle component. Participants excluded due to missing data were more likely to be older and female and had higher HbA1c levels than those included in the study (Table S2).

### 2.2 Assessment of SDHs

The Healthy People 2030 Initiative (https://health.gov/healthypeople) proposed a framework encompassing five domains of SDHs: economic stability, education access and quality, health care access and quality, neighbourhood and built environment, and social and community context. We selected 17 variables to assess SDHs aligning with the Healthy People 2030 objectives and previous UK Biobank‐based relevant studies.^16^, ^17^ Briefly, household income (pre‐tax, <£31 000), employment status, and income score (indicator of area‐level income deprivation) were included to assess financial circumstances. Educational attainment and education score (indicator of area‐level deprivation related to education, training and skills) were included to assess education access and quality. Health score (indicator of area‐level healthcare deprivation) was included to assess health care access and quality. House ownership, crime score (indicator of area‐level major crime type incidence), housing score (indicator of area‐level physical and financial accessibility of housing and key local services), and natural environment (percentage of home location data buffered at 1 km) were included to assess neighbourhood and built environment. Psychosocial problems, race/ethnicity, living alone, social support, social activity, social isolation and emotional distress were included to assess social and community context. All variables were collected using touchscreen questionnaires at baseline or via links to a multiple deprivation score index across each constituent nation of the UK. The detailed definitions were provided in Table S3.^16^ Each SDH was dichotomized into advantaged (scored 0) or disadvantaged level (scored 1). The overall SDH score was calculated by summing the scores of the 17 variables; the sums ranged from 0 to 17, with higher scores indicating greater social vulnerability. According to the SDH score tertiles, participants were classified as having favourable (scored 0–5), medium (scored 6–8) or unfavourable SDHs (scored 9–17).

### 2.3 Follow‐up for CRMDs and death

FCRMD was defined as the first occurrence of any CRMD (CVD, T2D or CKD) during follow‐up.^6^, ^23^, ^24^ CRMM was defined as at least two coexisting CRMDs after FCRMD.^6^, ^23^ The UK Biobank ascertained the incidence of CRMDs through self‐reported, primary care and hospital admission sources. The data were available until 31 October 2022, 31 May 2022 and 31 August 2022 for England, Wales and Scotland, respectively.^25^

Death information was ascertained via links to the National Health Service (NHS) Information Centre (England and Wales) and NHS Central Register (Scotland) death registries and was available up to 30 November 2022. In this study, follow‐up was censored at death, loss to follow‐up, or the final date with available data, whichever came first. CVD included coronary heart disease, stroke, heart failure, atrial fibrillation, and peripheral artery disease. CRMD diagnoses were ascertained using the corresponding International Classification of Diseases 10th revision (ICD‐10) and/or the Office of Population Censuses and Surveys Classification of Interventions and Procedures, version 4 codes. Table S4 documents ascertainment details.

### 2.4 Assessment of lifestyle and covariates

In line with previous studies, we constructed a healthy lifestyle score integrating five modifiable behavioural factors (diet, smoking, alcohol drinking, physical activity and sleep).^17^, ^26^ Dietary quality was assessed based on recent dietary recommendations for cardiovascular health, which consider 10 food groups (adequate intake of fruits, vegetables, whole grains, fish/shellfish, dairy, vegetable oils and refined grains and reduced intake of processed meats, unprocessed meats and sugar‐sweetened beverages). A healthy diet was defined as meeting five or more recommendations.^27^ Participants were asked about their smoking status and classified as never smoker or not. No excessive drinking (including alcohol abstinence or drinking in moderation) was defined as daily alcohol intake of ≤1 drink for women and ≤2 drinks for men.^28^ Healthy physical activity was defined as at least 150 min of moderate activity or 75 min of vigorous activity per week (or an equivalent combination).^29^ Healthy sleep was defined as a daily sleep duration of 7–8 h.^30^ For each lifestyle factor, the healthy condition (i.e., healthy diet, never smoking, alcohol abstinence or drinking in moderation, healthy physical activity and healthy sleep) was scored 1, and the unhealthy condition was scored 0. The overall lifestyle score was calculated by summing the scores of the five components ranging from 0 to 5, with higher score indicating better adherence to healthy lifestyle. Participants were classified as favourable (scored 4–5, *n* = 8407), medium (scored 3, *n* = 13 271), or unfavourable (scored 0–2, *n* = 15 420) by tertiles. Age, sex, healthy body mass index (BMI; ≥18.5 and <25 kg/m^2^), hypertension prevalence and HbA1c concentration were considered as covariates.

### 2.5 Statistical analyses

We examined the associations of different SDH levels with each state (i.e., FCRMD, CRMM and death) using a Cox proportional hazards model. No significant violation of the proportional hazards assumption was observed using scaled Schoenfeld residual plots.^31^

Subsequently, we used the multistate model to assess the associations between different SDH levels and temporal disease progression from prediabetes to FCRMD, CRMM and death. The multistate model, performed with Markov proportional hazards, is an extension of the competing risk survival model. It encompasses more than two disease states and transitions, enabling estimation of the effects of a certain factor in different stages of a disease process.^32^, ^33^ It has been widely used in epidemiological studies investigating multimorbidity progression.^23^, ^34^, ^35^, ^36^

**FIGURE 1:**
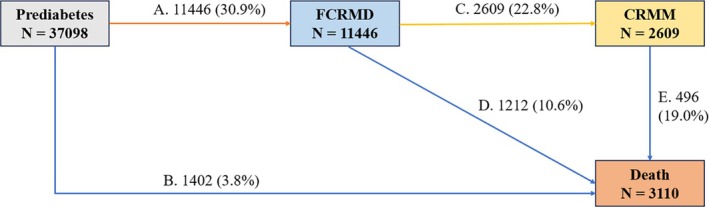
Numbers (percentages) of participants in transition pattern A from prediabetes to first cardio–renal–metabolic disease (FCRMD), cardio–renal–metabolic multimorbidity (coexistence of two or three cardio–renal–metabolic diseases) (CRMM) and death.

Five transition paths were constructed based on the natural history of prediabetes progression and previous studies (transition pattern A; Figure 1): I, prediabetes to FCRMD; II, prediabetes to death; III, FCRMD to CRMM; IV, FCRMD to death; and V, CRMM to death. For participants with identical entry dates for different states, we assigned the entry date of the theoretical former state as that of the latter state minus 0.5 day. Two multivariable adjusted models were constructed: model 1 was adjusted for age, sex, BMI status, prevalence of hypertension and HbA1c; model 2 was further adjusted for overall lifestyle score levels. We tested the rationale of assuming lifestyle as potential mediators (i.e., lifestyle showed significant associations with both SDHs and progression outcomes); details were provided in the Methods, Supporting Information S1. The difference method was used to examine the mediation proportion by lifestyle for the associations between SDHs and each outcome by comparing estimates from models with and without the hypothesised mediator variable.^26^, ^37^ We also predicted the transition probabilities from one state to the next during follow‐up using the multistate model. Probabilities were separately estimated for participants with favourable and unfavourable SDH levels, with values of each covariate set at the average level among the population. Paired sample *t* tests were performed to compare the probabilities at the two SDH levels.

**FIGURE 2:**
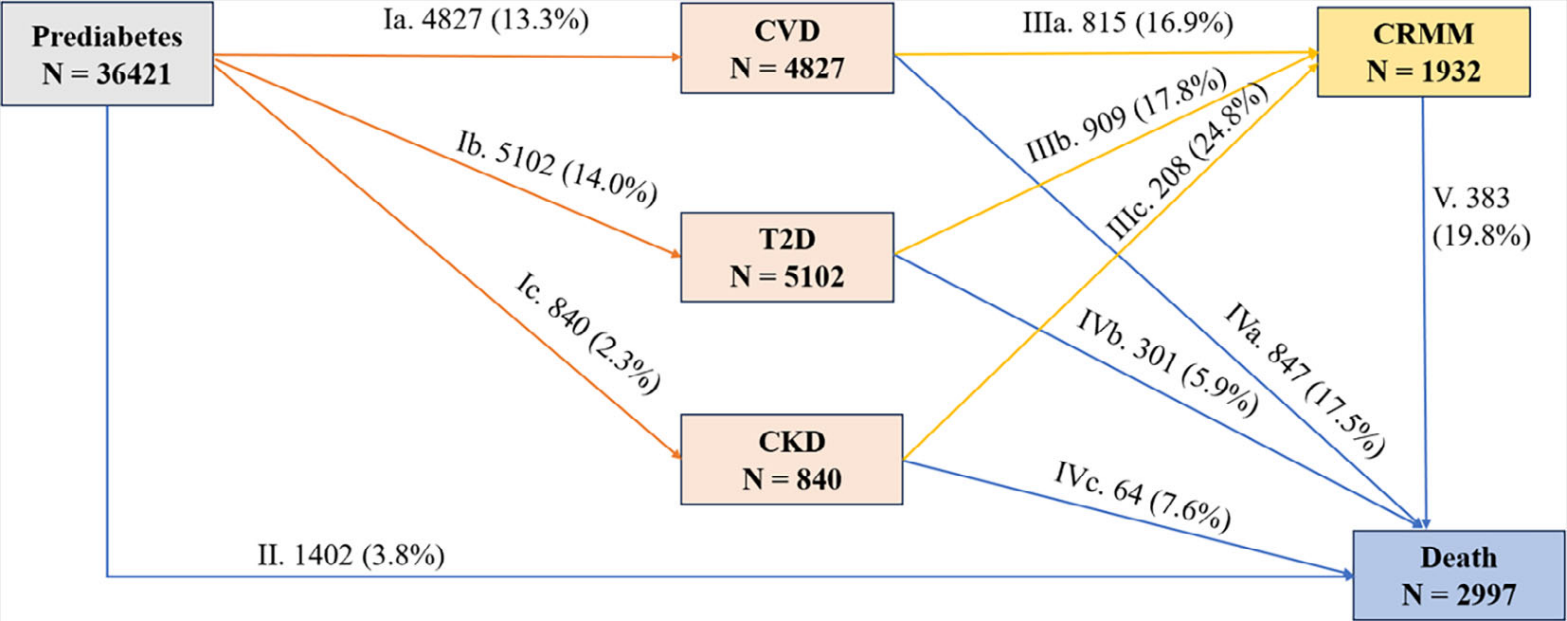
Numbers (percentages) of participants in transition pattern B from prediabetes to specific first cardio–renal–metabolic disease, cardio–renal–metabolic multimorbidity (coexistence of two or three cardio–renal–metabolic diseases [CRMDs]) (CRMM), and death. CRMDs include cardiovascular disease (CVD), type 2 diabetes (T2D), and chronic kidney disease (CKD).

To explore the disease‐specific associations, we extended pattern A by separately investigating FCRMD as T2D, CVD and CKD (transition pattern B; Figure 2). Accordingly, six states were included and 11 transition paths were considered. To determine the temporal sequence of incident individual disease, we excluded 677 (1.8%) participants who were diagnosed with two or three of the CRMDs on the same date, leaving 36 421 participants in this analysis. The multistate model was adjusted for all covariates, including the overall lifestyle score.

### 2.6 Sensitivity analyses

Several sensitivity analyses were conducted to examine the robustness of our findings regarding transition pattern A. First, we excluded participants who had clinical events within the first 2 years of follow‐up to reduce the risk of potential reverse causality. Second, we further adjusted for drug use (i.e., use of antihypertensive drugs and statins) to reduce potential confounding bias. Third, we further adjusted for cardiovascular and renal biomarkers (i.e., serum low‐density lipoprotein cholesterol [LDL‐C] and eGFR). Fourth, for participants entering different stages on the same day, we introduced different time intervals (1, 30 and 365 days) to evaluate the potential effects of these intervals. Fifth, to examine potential effect modifiers, we performed stratified analyses according to sex and age (<60 or ≥60 years). Interactions were tested using the likelihood ratio test, which compared models with and without a cross‐product term. Sixth, to evaluate the robustness of our mediation results to the mediator's categorisation, we conducted a sensitivity analysis treating the overall lifestyle score as a continuous variable.

Based on pattern B, we focused on different CRMM combinations (Figure S2). Four states (CVD–T2D multimorbidity, CVD–CKD multimorbidity, T2D–CKD multimorbidity and three CRMMs) were included, and 12 paths were considered.^23^

All analyses were performed using R 4.2.1 (R Foundation, Vienna, Austria) and SAS 9.4 (SAS Institute Inc., Cary, NC, USA). A two‐sided *p* value <0.05 was considered statistical significance.

## 3 RESULTS

### 3.1 Characteristics

We included 37 098 participants (mean age: 58.8 ± 7.1 years, 54.1% women) with prediabetes (Table 1), of whom 11 786 (31.7%), 13 567 (36.6%) and 11 745 (31.7%) were categorised into the favourable, medium, and unfavourable SDH groups, respectively. The unfavourable SDH group participants were more likely to be younger and female and have a higher BMI, HbA1c and prevalence of hypertension and unhealthier lifestyle factors. Table S5 shows the proportions of participants with disadvantaged levels for each SDH item.

**TABLE 1:** Baseline characteristics of participants included in the current analysis.

Characteristic	Overall (*n* = 37 098)	Favourable SDHs (*n* = 11 786)	Medium SDHs (*n* = 13 567)	Unfavourable SDHs (*n* = 11 745)	*p* for trend^a^
Age (years)	58.8 ± 7.1	60.0 ± 6.5	59.0 ± 7.0	57.3 ± 7.5	<0.001
Female (%)	20 067 (54.1)	6235 (52.9)	7391 (54.5)	6441 (54.8)	0.003
BMI (kg/m^2^)	28.8 ± 5.3	28.0 ± 4.7	28.8 ± 5.2	29.8 ± 5.8	<0.001
Healthy BMI (%)	8429 (22.7)	3196 (27.1)	3042 (22.4)	2191 (18.7)	<0.001
Hypertension (%)	11 870 (32.0)	3481 (29.5)	4383 (32.3)	4006 (34.1)	<0.001
HbA1c (mmol/mol)	41.1 ± 2.0	41.0 ± 1.9	41.1 ± 2.0	41.3 ± 2.1	<0.001
Lifestyle score	3 (2, 3)	3 (2, 4)	3 (2, 3)	2 (2, 3)	<0.001
Healthy diet (%)	5237 (14.1)	1759 (14.9)	1886 (13.9)	1592 (13.6)	0.003
Never smoking (%)	18 504 (49.9)	6690 (56.8)	6774 (49.9)	5040 (42.9)	<0.001
No excessive drinking (%)	32 165 (86.7)	10 462 (88.8)	11 723 (86.4)	9980 (85.0)	<0.001
Healthy sleep (%)	24 125 (65.0)	8454 (71.7)	8985 (66.2)	6686 (56.9)	<0.001
Healthy physical activity (%)	20 312 (54.8)	6969 (59.1)	7425 (54.7)	5918 (50.4)	<0.001

*Note*: Healthy BMI was defined as a BMI ≥18.5 and < 25 kg/m^2^. A healthy diet was defined as meeting five or more of 10 dietary intake recommendations. No excessive drinking included alcohol abstinence or drinking in moderation (daily alcohol intake of ≤1 drink for women and ≤2 drinks for men). Healthy sleep was defined as daily sleep duration of 7 or 8 h. Healthy physical activity was defined as at least 150 min of moderate activity or 75 min of vigorous activity (or an equivalent combination).

Abbreviations: BMI, body mass index; HbA1c, haemoglobin; SDHs, social determinants of health.

^a^ Calculated using linear regression analysis or chi‐square test for trend.

As shown in Figure 1, at a mean follow‐up of 13.7 (interquartile range: 13.0, 14.4) years, 11 446 (transition I, 30.9%) participants developed the first CRMD, while 1402 (transition II, 3.8%) directly died from all causes. Among those who experienced the first CRMD, 2609 (transition III, 22.8%) developed CRMM, of whom 496 (transition V, 19.0%) subsequently died, while 1212 (transition IV, 10.6%) died without developing CRMM. As for disease‐specific transitions (677 participants excluded, 36 421 participants included), 4827 (transition Ia, 13.3%) first experienced CVD, 5102 (transition Ib, 14.0%) first experienced T2D and 840 (transition Ic, 2.3%) first experienced CKD (Figure 2).

### 3.2 SDHs and longitudinal progression in transition pattern A

In conventional Cox models, both the medium and unfavourable SDH groups showed higher risks of incident FCRMD, CRMM and death than the favourable SDH group (Table S6).

Table 2 shows the multistate model analysis of the associations between SDH levels and prediabetes progression (transition pattern A). Model 2, adjusted for the lifestyle score and other covariates, revealed that, compared with the favourable SDH group, the unfavourable SDH group showed a hazard ratio (HR) of 1.32 (95% confidence interval [CI]: 1.26–1.38, *p* < 0.001) for prediabetes‐to‐FCRMD transition and had higher risks of prediabetes‐to‐death (HR = 1.39, 95% CI: 1.22–1.59, *p* < 0.001), FCRMD‐to‐CRMM (HR = 1.35, 95% CI: 1.22–1.49, *p* < 0.001) and FCRMD‐to‐death transition (HR = 1.56, 95% CI: 1.35–1.81, *p* < 0.001). However, unfavourable SDHs were not significantly associated with an increased risk of CRMM‐to‐death transition (HR = 1.16, 95% CI: 0.92–1.47, *p* = 0.210).

**TABLE 2:** Association between social determinants of health (SDH) level and progressions from prediabetes in transition pattern A using the multistate model.

SDH level	No. of events	Model 1		Model 2		Mediation proportion (%) (95% CI)
		HR (95% CI)	*p*	HR (95% CI)	*p*	
Prediabetes to FCRMD						
Favourable	3198	Reference	–	Reference	–	
Medium	4143	1.14 (1.09, 1.19)	<0.001	1.12 (1.07, 1.17)	<0.001	18.1 (11.2, 28.0)
Unfavourable	4105	1.36 (1.30, 1.43)	<0.001	1.32 (1.26, 1.38)	<0.001	14.0 (11.1, 17.6)
Prediabetes to death						
Favourable	419	Reference	–	Reference	–	
Medium	504	1.16 (1.02, 1.33)	0.022	1.12 (0.99, 1.28)	0.078	25.1 (8.4, 55.1)
Unfavourable	479	1.50 (1.31, 1.71)	< 0.001	1.39 (1.22, 1.59)	< 0.001	19.2 (12.4, 28.5)
FCRMD to CRMM						
Favourable	664	Reference	–	Reference	–	
Medium	934	1.13 (1.02, 1.25)	0.017	1.12 (1.01, 1.23)	0.031	13.1 (3.6, 38.3)
Unfavourable	1011	1.38 (1.24, 1.52)	< 0.001	1.35 (1.22, 1.49)	<0.001	8.8 (4.7, 16.1)
FCRMD to death						
Favourable	316	Reference	–	Reference	–	
Medium	407	1.12 (0.97, 1.30)	0.118	1.10 (0.95, 1.27)	0.225	–
Unfavourable	489	1.65 (1.43, 1.90)	<0.001	1.56 (1.35, 1.81)	<0.001	13.5 (8.0, 22.0)
CRMM to death						
Favourable	119	Reference	–	Reference	–	
Medium	184	1.18 (0.93, 1.48)	0.170	1.15 (0.91, 1.45)	0.240	–
Unfavourable	193	1.21 (0.96, 1.52)	0.114	1.16 (0.92, 1.47)	0.210	–

*Note*: Model 1 was adjusted for age, sex, healthy BMI status, prevalence of hypertension, and HbA1c. Model 2 was further adjusted for levels of overall lifestyle score. The mediation proportion of lifestyle was calculated when SDHs shown a significant association in Model 1, by using the difference methods which comparing the estimates of SDHs between Model 1 and Model 2.

Abbreviations: CI, confidence interval; CRMM, cardio–renal–metabolic multimorbidity; FCRMD, first cardio–renal–metabolic disease; HR, hazard ratio.

Lifestyle partially mediated the associations, with the largest mediation proportion of 19.2% (95% CI: 12.4%–28.5%) observed in the prediabetes‐to‐death transition, and the lowest mediation proportion of 8.8% (95% CI: 4.7%–16.1%) observed in the FCRMD‐to‐CRMM transition (Table 2).

Figure S3 presents the transition probabilities of the favourable and unfavourable SDH groups. The unfavourable SDH group disproportionately exhibited higher probabilities of each transition (all *p* < 0.001).

The medium SDH group showed significantly higher risks of prediabetes to FCRMD (HR = 1.12, 95% CI: 1.07–1.17, *p* < 0.001) and FCRMD to CRMM transition (HR = 1.12, 95% CI: 1.01–1.23, *p* = 0.031) than the favourable SDH group in the fully adjusted model (Table 2).

### 3.3 SDHs and longitudinal progression in transition pattern B

**FIGURE 3:**
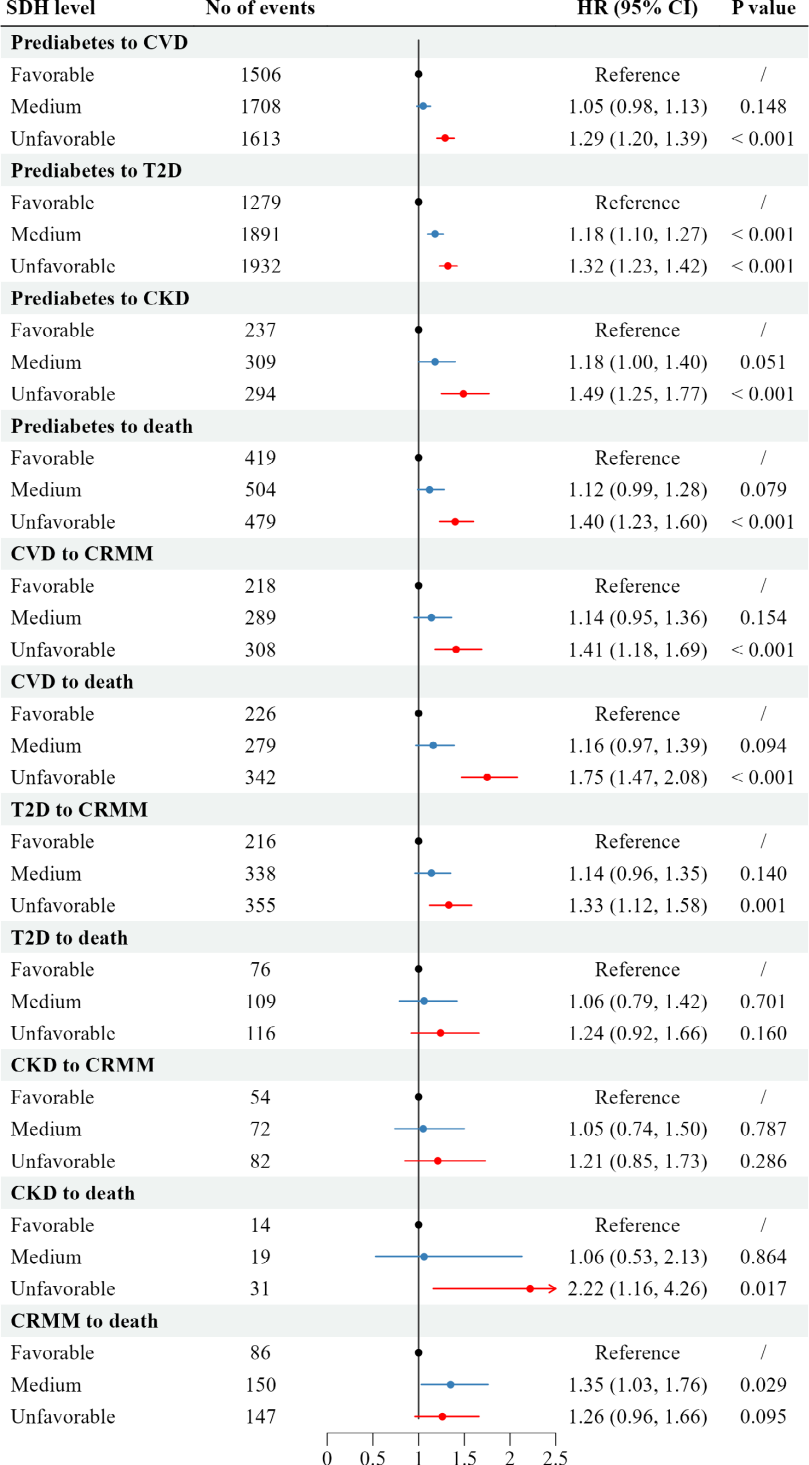
Association between the social determinants of health (SDH) level and progression from prediabetes in transition pattern B using the multistate model. The model was adjusted for age, sex, healthy body mass index status, prevalence of hypertension, HbA1c, and the overall lifestyle score level. CI, confidence interval; CKD, chronic kidney disease; CRMM, cardio–renal–metabolic multimorbidity; CVD, cardiovascular disease; HR, hazard ratio; T2D, type 2 diabetes.

Compared with the favourable SDH group, the HRs in the unfavourable SDH group were 1.29 (95% CI: 1.20–1.39, *p* < 0.001) for prediabetes‐to‐CVD transition, 1.32 (95% CI: 1.23–1.42, *p* < 0.001) for prediabetes‐to‐T2D transition, and 1.49 (95% CI: 1.25–1.77, *p* < 0.001) for baseline‐to‐CKD transition (Figure 3).

The unfavourable SDH group showed significantly higher risks of CVD to multimorbidity (HR = 1.41, 95% CI: 1.18–169, *p* < 0.001), CVD to death (HR = 1.75, 95% CI: 1.47–2.08, *p* < 0.001), and T2D to multimorbidity transition (HR = 1.33, 95% CI: 1.12–1.58, *p* = 0.001) than the favourable SDH group, with the highest risk for the CKD to death transition (HR = 2.22, 95% CI: 1.16–4.26, *p* = 0.017).

### 3.4 Sensitivity analyses

Our sensitivity analyses excluding 1707 events that occurred within the first 2 years of follow‐up (Table S7), further adjusting for drug use (Table S8), further adjusting for serum LDL‐C and eGFR (Table S9), and trying several alternative time intervals for participants who entered different states on the same day (Table S10) yielded generally consistent findings with the main analysis. Our stratified analyses (Table S11) revealed that sex and age modified the association of SDHs with prediabetes‐to‐FCRMD transition (*p*_interaction_ <0.001 and 0.020, respectively). The unfavourable SDH group showed a higher risk of prediabetes‐to‐FCRMD transition among women (HR = 1.51, 95% CI: 1.40–1.62) than among men (HR = 1.18, 95% CI: 1.11–1.26) and a higher risk among <60‐year‐old individuals (HR = 1.35, 95% CI: 1.24–1.46) than among ≥60‐year‐old individuals (HR = 1.25, 95% CI: 1.18–1.33). The mediation proportions of treating lifestyle score as a continuous variable were generally consistent with our main analysis (Table S12). The mediation proportions were slightly higher, while lifestyle still explained limited proportions (less than 25%) of the adverse effects associated with unfavourable SDHs at each stage.

Regarding specific multimorbidity combinations (Figure S2), the unfavourable SDH group showed significantly higher risks of CVD‐to‐CVD–T2D multimorbidity (HR = 1.42, 95% CI: 1.15–1.76, *p* = 0.001), CVD‐to‐CVD–CKD multimorbidity (HR = 1.43, 95% CI: 1.02–2.01, *p* = 0.037) and T2D‐to‐T2D–CVD multimorbidity transition (HR = 1.34, 95% CI: 1.11–1.63, *p* = 0.003) than the favourable SDH group (Figure S4).

## 4 DISCUSSION

This is the first study to evaluate the effects of combined SDHs on the longitudinal progression from prediabetes to CRMM and death, using data from a prospective UK Biobank cohort of over 37 000 adults with prediabetes. We found that the unfavourable SDH group had significantly elevated risks of transitioning from prediabetes to FCRMD and death and from FCRMD to CRMM and death. Lifestyle partially mediated these associations, with the largest mediation proportion (around one fifth) observed in the prediabetes‐to‐death transition. Among disease‐specific associations, unfavourable SDHs disproportionally increased the risks of three CRMDs, with the highest risk observed for the CKD‐to‐death transition. Our findings highlight the urgent need to implement health policies that mitigate socioeconomic disparity and promote lifestyle modifications in socially disadvantaged populations.

From a biological perspective, unfavourable SDHs act as persistent psychosocial and environmental stressors that aggravate allostatic load, contributing to metabolic dysregulation and chronic inflammation.^38^ They also contribute to limited accessibility to healthcare resources and poor adherence to healthy behaviours, resulting in unfavourable management of CRMD.^11^, ^39^, ^40^ The concept of a polysocial score that combines information about multifaceted SDHs has gained increasing attention.^15^ Using well‐characterised UK Biobank data, studies have found that a disadvantageous SDH level, determined by a combined SDH score, was significantly associated with increased risks of incident diabetes in the general population^17^ and incident morbidities (e.g., CVD and microvascular disease) in people with diabetes.^16^ Our study provides novel evidence regarding prediabetes, a subclinical state recognised as a critical time window for intervention. Routine screening for SDHs should be conducted alongside clinical assessment early during the diabetes care continuum to identify potential social risks and needs and inform personalised interventions to address disparity.

Using a multistate model depicting different transition paths, we delineated the effect of SDHs on the dynamic prediabetes‐to‐CRMM progression. Compared with the favourable SDH group, the unfavourable SDH group consistently showed elevated risks for different transition stages, except for the CRMM‐to‐death transition. These findings suggest that individuals with unfavourable SDHs should be screened, followed by intervention before a more irreversible advanced stage occurs (i.e., progression to CRMM). Furthermore, we found that adherence to ideal lifestyle factors (i.e., healthy diet, smoking cessation, alcohol abstinence or drinking in moderation, regular physical activity, and healthy sleep) attenuated the adverse effects of unfavourable SDHs, supporting the importance of intensive lifestyle modification in resource‐limited areas. However, the mediation proportions at each stage explained less than one fifth of the effects. The limited mediation effects of lifestyle were consistent with previous studies,^26^, ^41^ underscoring that health disparities rooted in social inequities cannot be eliminated by promoting healthy lifestyle alone. Screening and care services for socially vulnerable communities should be prioritised. Populational‐level multidimensional and sustainable policies and programmes, such as Affordable Care Act Medicaid expansion and the Community Health Worker Programmes, are as important.^42^, ^43^ The mediation proportions seemed larger in progressions from prediabetes than in progressions from FCRMD, reiterating the importance of early prevention. Our stratified analyses also showed that women and middle‐to‐old adults (<60 years old) had higher unfavourable‐SDH‐related risks of progressing from prediabetes to FCRMD than their counterparts; these subpopulations are likely to be more vulnerable to social burdens attributed to pressure from career and family and warrant targeted intervention. Our findings align with previous findings that the combined SDH score can serve as a screening tool and that providing support for individuals with disadvantaged SDHs should be recognised as an important principle throughout the diabetes care continuum.

In the disease‐specific associations (Pattern B), we found that unfavourable SDHs were associated with increased risks of first progressing to CVD, T2D or CKD and further progressing to CVD–T2D multimorbidity. These findings were consistent with previous studies showing adverse effects of unfavourable SDHs on individual CRMDs and cardiometabolic multimorbidity.^11^, ^12^, ^17^, ^39^ The highest risk (HR = 2.22) posed by unfavourable SDHs was observed for the first CKD‐to‐death transition. The magnitude of hazards for incident CKD (HR = 1.49) also seemed larger than that for incident CVD (HR = 1.29) or T2D (HR = 1.32). As the decline in kidney function is gradual and may initially present no symptoms, the latter pathophysiological spectrum of CKD is usually irreversible, especially with existing comorbidities like prediabetes.^44^, ^45^ The awareness, diagnosis, and treatment rates were lower in CKD management than in T2D and CVD management in which routine prevention and care (e.g., accessible educational programmes, specialty clinics, first‐line and second‐line medications) have been better integrated into the healthcare system.^40^, ^44^ Individuals with unfavourable SDHs may be particularly susceptible to CKD progression; for example, studies have demonstrated that CKD patients with unfavourable SDHs progressed to kidney failure in a less optimal manner (i.e., longer wait times for kidney transplantation, starting dialysis in the inpatient rather than outpatient setting, and initiating dialysis without a pre‐created access) than those with favourable SDHs.^18^, ^46^ Our study ascertained CKD through ICD‐10th codes, probably capturing more severe cases and underestimating the incidence of CKD. Nevertheless, our findings revealed that the pronounced risks of CKD and its progression in people with prediabetes should not be ignored, especially among those with unfavourable SDHs. Further research with a larger sample size and comprehensive renal biomarker measurements is warranted to validate our findings.

This study has several strengths. This is the first comprehensive study of evaluating how combined SDHs could affect the dynamic progression from prediabetes to FCRMD, CRMM and mortality, as well as examining contributions of lifestyle as potential mediators. In particular, we leveraged the multistate framework to provide a holistic and life‐course perspective of the disease continuum. Moreover, the systematic assessment of SDHs, large sample size, and long follow‐up period ensured the robustness of our findings. However, some limitations should be noted. First, due to data availability, we identified people with prediabetes using HbA1c, although prediabetes has alternative definitions based on fasting plasma glucose levels and the oral glucose tolerance test. Future studies using different criteria to ascertain prediabetes are needed to examine the robustness of our findings. Second, the AHA recently proposed the cardiovascular–kidney–metabolic (CKM) syndrome, a multiorgan disorder depicting five stages across the whole pathophysiological spectrum.^8^ However, we were unable to account for the full progression of CKM syndrome, as certain stages involved subclinical progressions (e.g., coronary artery calcification, abnormalities of myocardial structure and function) which were not assessed during follow‐up. Nevertheless, our study aligns with AHA's recommendation for integrated, equity‐focused care of CKM conditions. Third, although we carefully considered the different aspects of SDHs, the combined score may not have accurately quantified the SDH level or fully accounted for the intrinsic interactions across social factors. A well‐established SDH score validated in different settings is still warranted. Additionally, SDHs and covariates were assessed only at baseline, and some data were collected through self‐reports, which may have led to misclassification and biassed findings towards null associations. Fourth, causality could not be established due to the observational nature of the study, although various sensitivity analyses were conducted to reduce the risk of reverse causality and residual confounding. Finally, health volunteer bias may have occurred, that is, people who did not enrol in the UK Biobank may be more likely to have had less favourable SDH levels and unhealthier conditions,^47^ which might have caused underestimation in our results. Further, the UK Biobank samples were predominantly from Caucasians aged 40 to 69, which limited the generalizability of our findings to populations with different demographic characteristics.

In conclusion, we found that unfavourable SDHs were associated with increased risks of progressing from prediabetes to FCRMD, CRMM, and death. The findings suggest that resource allocation and lifestyle promotion be prioritised for those with unfavourable SDHs to mitigate disparities in progression to CRMM and death in diabetes care, especially in the early disease stage.

## AUTHOR CONTRIBUTIONS

Design: **RH**, **YTC**. Conduct/data collection: **RH**. Analysis: **RH**. Writing manuscript: **RH**, **AY**, **LG**, **EC**, **WJ**, **YTC**.

## FUNDING INFORMATION

No funding was received for this study.

## CONFLICT OF INTEREST STATEMENT

The authors declare that they have no competing interests.

## PEER REVIEW

The peer review history for this article is available at https://www.webofscience.com/api/gateway/wos/peer‐review/10.1111/dom.70067.

## Supporting information


**Data S1.** Supporting Information.

## Data Availability

The datasets generated and/or analysed during the current study derive from the UK Biobank Resource under Application Number 300879. The UK Biobank datasets are available after application (https://www.ukbiobank.ac.uk/enable-your-research/apply-for-access).

## References

[1] Rooney MR , Fang M , Ogurtsova K , et al. Global prevalence of prediabetes. Diabetes Care. 2023;46(7):1388‐1394. doi:10.2337/dc22-2376 37196350 PMC10442190

[2] Rooney MR , Wallace AS , Echouffo Tcheugui JB , et al. Prediabetes is associated with elevated risk of clinical outcomes even without progression to diabetes. Diabetologia. 2024;68:357‐366. doi:10.1007/s00125-024-06315-0 39531040 PMC11732724

[3] Zhang X , Wu H , Fan B , et al. The role of age on the risk relationship between prediabetes and major morbidities and mortality: analysis of the Hong Kong diabetes surveillance database of 2 million Chinese adults. Lancet Reg Health West Pac. 2023;30:100599. doi:10.1016/j.lanwpc.2022.100599 36419741 PMC9677132

[4] Ndumele CE , Neeland IJ , Tuttle KR , et al. A synopsis of the evidence for the science and clinical management of cardiovascular‐kidney‐metabolic (CKM) syndrome: a scientific statement from the American Heart Association. Circulation. 2023;148(20):1636‐1664. doi:10.1161/cir.0000000000001186 37807920

[5] Ferrari AJ , Santomauro DF , Aali A , et al. Global incidence, prevalence, years lived with disability (YLDs), disability‐adjusted life‐years (DALYs), and healthy life expectancy (HALE) for 371 diseases and injuries in 204 countries and territories and 811 subnational locations, 1990–2021: a systematic analysis for the global burden of disease study 2021. Lancet. 2024;403(10440):2133‐2161. doi:10.1016/S0140-6736(24)00757-8 38642570 PMC11122111

[6] Ostrominski JW , Arnold SV , Butler J , et al. Prevalence and overlap of cardiac, renal, and metabolic conditions in US adults, 1999‐2020. JAMA Cardiol. 2023;8(11):1050‐1060. doi:10.1001/jamacardio.2023.3241 37755728 PMC10535010

[7] Cherney DZI , Repetto E , Wheeler DC , et al. Impact of cardio‐renal‐metabolic comorbidities on cardiovascular outcomes and mortality in type 2 diabetes mellitus. Am J Nephrol. 2020;51(1):74‐82. doi:10.1159/000504558 31812955

[8] Ndumele CE , Rangaswami J , Chow SL , et al. Cardiovascular‐kidney‐metabolic health: a presidential advisory from the American Heart Association. Circulation. 2023;148(20):1606‐1635. doi:10.1161/cir.0000000000001184 37807924

[9] Gong Q , Zhang P , Wang J , et al. Morbidity and mortality after lifestyle intervention for people with impaired glucose tolerance: 30‐year results of the Da Qing diabetes prevention outcome study. Lancet Diabetes Endocrinol. 2019;7(6):452‐461. doi:10.1016/s2213-8587(19)30093-2 31036503 PMC8172050

[10] Lee CG , Heckman‐Stoddard B , Dabelea D , et al. Effect of metformin and lifestyle interventions on mortality in the diabetes prevention program and diabetes prevention program outcomes study. Diabetes Care. 2021;44(12):2775‐2782. doi:10.2337/dc21-1046 34697033 PMC8669534

[11] Hill‐Briggs F , Adler NE , Berkowitz SA , et al. Social determinants of health and diabetes: a scientific review. Diabetes Care. 2020;44(1):258‐279. doi:10.2337/dci20-0053 33139407 PMC7783927

[12] Safford MM , Reshetnyak E , Sterling MR , et al. Number of social determinants of health and fatal and nonfatal incident coronary heart disease in the REGARDS study. Circulation. 2021;143(3):244‐253. doi:10.1161/circulationaha.120.048026 33269599 PMC7856168

[13] Agardh E , Allebeck P , Hallqvist J , Moradi T , Sidorchuk A . Type 2 diabetes incidence and socio‐economic position: a systematic review and meta‐analysis. Int J Epidemiol. 2011;40(3):804‐818. doi:10.1093/ije/dyr029 21335614

[14] Berkowitz SA , Baggett TP , Wexler DJ , Huskey KW , Wee CC . Food insecurity and metabolic control among U.S. adults with diabetes. Diabetes Care. 2013;36(10):3093‐3099. doi:10.2337/dc13-0570 23757436 PMC3781549

[15] Figueroa JF , Frakt AB , Jha AK . Addressing social determinants of health: time for a polysocial risk score. JAMA. 2020;323(16):1553‐1554. doi:10.1001/jama.2020.2436 32242887

[16] Zhong J , Zhang Y , Zhu K , et al. Associations of social determinants of health with life expectancy and future health risks among individuals with type 2 diabetes: two nationwide cohort studies in the UK and USA. Lancet Healthy Longev. 2024;5(8):e542‐e551. doi:10.1016/s2666-7568(24)00116-8 39106873

[17] Zhao Y , Li Y , Zhuang Z , et al. Associations of polysocial risk score, lifestyle and genetic factors with incident type 2 diabetes: a prospective cohort study. Diabetologia. 2022;65(12):2056‐2065. doi:10.1007/s00125-022-05761-y 35859134

[18] Hundemer GL , Ravani P , Sood MM , et al. Social determinants of health and the transition from advanced chronic kidney disease to kidney failure. Nephrol Dial Transplant. 2023;38(7):1682‐1690. doi:10.1093/ndt/gfac302 36316015 PMC10310519

[19] Sudlow C , Gallacher J , Allen N , et al. UK Biobank: an open access resource for identifying the causes of a wide range of complex diseases of middle and old age. PLoS Med. 2015;12(3):e1001779. doi:10.1371/journal.pmed.1001779 25826379 PMC4380465

[20] Vandenbroucke JP , von Elm E , Altman DG , et al. Strengthening the reporting of observational studies in epidemiology (STROBE): explanation and elaboration. PLoS Med. 2007;4(10):e297. doi:10.1371/journal.pmed.0040297 17941715 PMC2020496

[21] ElSayed NA , Aleppo G , Aroda VR , et al. 2. Classification and diagnosis of diabetes: standards of care in diabetes‐2023. Diabetes Care. 2023;46(Suppl 1):S19‐s40. doi:10.2337/dc23-S002 36507649 PMC9810477

[22] Huang Y , Zhang Y , Zhou C , et al. Association of dietary manganese intake with new‐onset chronic kidney disease in participants with diabetes. Diabetes Metab Syndr. 2024;18(10):103138. doi:10.1016/j.dsx.2024.103138 39413577

[23] Zhang N , Liu X , Wang L , et al. Lifestyle factors and their relative contributions to longitudinal progression of cardio‐renal‐metabolic multimorbidity: a prospective cohort study. Cardiovasc Diabetol. 2024;23(1):265. doi:10.1186/s12933-024-02347-3 39026309 PMC11264843

[24] Marassi M , Fadini GP . The cardio‐renal‐metabolic connection: a review of the evidence. Cardiovasc Diabetol. 2023;22(1):195. doi:10.1186/s12933-023-01937-x 37525273 PMC10391899

[25] Biobank TU . Data providers and dates of data availability. https://biobank.ndph.ox.ac.uk/showcase/exinfo.cgi?src=Data_providers_and_dates. Accessed 1 May 2025

[26] Zhang YB , Chen C , Pan XF , et al. Associations of healthy lifestyle and socioeconomic status with mortality and incident cardiovascular disease: two prospective cohort studies. BMJ. 2021;373:n604. doi:10.1136/bmj.n604 33853828 PMC8044922

[27] Said MA , Verweij N , van der Harst P . Associations of combined genetic and lifestyle risks with incident cardiovascular disease and diabetes in the UK Biobank study. JAMA Cardiol. 2018;3(8):693‐702. doi:10.1001/jamacardio.2018.1717 29955826 PMC6143077

[28] DeSalvo KB , Olson R , Casavale KO . Dietary guidelines for Americans. JAMA. 2016;315(5):457‐458. doi:10.1001/jama.2015.18396 26746707

[29] Lloyd‐Jones DM , Hong Y , Labarthe D , et al. Defining and setting national goals for cardiovascular health promotion and disease reduction: the American Heart Association's strategic impact goal through 2020 and beyond. Circulation. 2010;121(4):586‐613. doi:10.1161/circulationaha.109.192703 20089546

[30] Wang C , Bangdiwala SI , Rangarajan S , et al. Association of estimated sleep duration and naps with mortality and cardiovascular events: a study of 116 632 people from 21 countries. Eur Heart J. 2019;40(20):1620‐1629. doi:10.1093/eurheartj/ehy695 30517670 PMC6528160

[31] Stensrud MJ , Hernán MA . Why test for proportional hazards? JAMA. 2020;323(14):1401‐1402. doi:10.1001/jama.2020.1267 32167523 PMC11983487

[32] Putter H , Fiocco M , Geskus RB . Tutorial in biostatistics: competing risks and multi‐state models. Stat Med. 2007;26(11):2389‐2430. doi:10.1002/sim.2712 17031868

[33] de Wreede LC , Fiocco M , Putter H . The mstate package for estimation and prediction in non‐ and semi‐parametric multi‐state and competing risks models. Comput Methods Programs Biomed. 2010;99(3):261‐274. doi:10.1016/j.cmpb.2010.01.001 20227129

[34] Li C , He D , Yang C , Zhang L . Daytime napping, incident atrial fibrillation, and dynamic transitions with dementia. JACC Adv. 2024;3(8):101108. doi:10.1016/j.jacadv.2024.101108 39105122 PMC11299576

[35] Guo H , Wang S , Peng H , et al. Life's essential 8 and cardiovascular diseases progression among adults in the United Kingdom. Metabolism. 2025;162:156031. doi:10.1016/j.metabol.2024.156031 39265807

[36] Han Y , Hu Y , Yu C , et al. Lifestyle, cardiometabolic disease, and multimorbidity in a prospective Chinese study. Eur Heart J. 2021;42(34):3374‐3384. doi:10.1093/eurheartj/ehab413 34333624 PMC8423468

[37] Nevo D , Liao X , Spiegelman D . Estimation and inference for the mediation proportion. Int J Biostat. 2017;13(2). doi:10.1515/ijb‐2017‐0006 10.1515/ijb-2017-0006 PMC6014631 28930628

[38] Guidi J , Lucente M , Sonino N , Fava GA . Allostatic load and its impact on health: a systematic review. Psychother Psychosom. 2021;90(1):11‐27. doi:10.1159/000510696 32799204

[39] Powell‐Wiley TM , Baumer Y , Baah FO , et al. Social determinants of cardiovascular disease. Circ Res. 2022;130(5):782‐799. doi:10.1161/circresaha.121.319811 35239404 PMC8893132

[40] Hall YN . Social determinants of health: addressing unmet needs in nephrology. Am J Kidney Dis. 2018;72(4):582‐591. doi:10.1053/j.ajkd.2017.12.016 29548780

[41] Bonaccio M , Di Castelnuovo A , Costanzo S , et al. Interaction between education and income on the risk of all‐cause mortality: prospective results from the MOLI‐SANI study. Int J Public Health. 2016;61(7):765‐776. doi:10.1007/s00038-016-0822-z 27091201

[42] Donohue JM , Cole ES , James CV , Jarlenski M , Michener JD , Roberts ET . The US Medicaid program: coverage, financing, reforms, and implications for health equity. JAMA. 2022;328(11):1085‐1099. doi:10.1001/jama.2022.14791 36125468

[43] Javanparast S , Windle A , Freeman T , Baum F . Community health worker programs to improve healthcare access and equity: are they only relevant to low‐ and middle‐income countries? Int J Health Policy Manag. 2018;7(10):943‐954. doi:10.15171/ijhpm.2018.53 30316247 PMC6186464

[44] Francis A , Harhay MN , Ong ACM , et al. Chronic kidney disease and the global public health agenda: an international consensus. Nat Rev Nephrol. 2024;20(7):473‐485. doi:10.1038/s41581-024-00820-6 38570631

[45] Zoccali C , Vanholder R , Massy ZA , et al. The systemic nature of CKD. Nat Rev Nephrol. 2017;13(6):344‐358. doi:10.1038/nrneph.2017.52 28435157

[46] Nicholas SB , Kalantar‐Zadeh K , Norris KC . Socioeconomic disparities in chronic kidney disease. Adv Chronic Kidney Dis. 2015;22(1):6‐15. doi:10.1053/j.ackd.2014.07.002 25573507 PMC4291541

[47] Fry A , Littlejohns TJ , Sudlow C , et al. Comparison of sociodemographic and health‐related characteristics of UK Biobank participants with those of the general population. Am J Epidemiol. 2017;186(9):1026‐1034. doi:10.1093/aje/kwx246 28641372 PMC5860371

