# Supplementary material for: Social determinants of health and progression to cardio–renal–metabolic multimorbidity and mortality in people with prediabetes: A prospective cohort study
Source: Diabetes Obes Metab. 2025 Aug 27;27(11):6605–14. doi: 10.1111/dom.70067 (PMC12515768; doi:10.1111/dom.70067)

**Supplemental Material**

**Supplemental Methods**

**Supplemental Table 1.** Missing numbers and percentages of each SDHs and lifestyle component.

**Supplement Table 2.** Comparison of characteristics between participants who were excluded due to missing data on any component of SDHs or lifestyle versus who were analyzed.

**Supplemental Table 3.** Assessment of SDHs in the UK Biobank.

**Supplemental Table 4.** International Classification of Disease and procedure codes used to ascertain incident outcomes.

**Supplemental Table 5.** Proportions of participants with disadvantaged levels for each SDHs item.

**Supplemental Table 6**. Associations of SDH levels with risks of FCRMD, CRMM, and death estimated by the Cox model.

**Supplemental Table 7.** Association between SDH level and progressions from prediabetes in the transition pattern A, after excluded participants (n = 1707) occurred the outcomes in the first two-years of follow-up.

**Supplemental Table 8.** Association between SDH level and progressions from prediabetes in the transition pattern A using multistate model further adjusting for drug use.

**Supplemental Table 9.** Association between SDH level and progressions from prediabetes in the transition pattern A using multistate model further adjusting for serum LDL-C and eGFR.

**Supplemental Table 10.** Association between SDH level and progressions from prediabetes in the transition pattern A, using different intervals for the participants entering different states on the same day.

**Supplemental Table 11.** Association between SDH level and progressions from prediabetes in the transition pattern A, stratified analyses by sex and age group.

**Supplemental Table 12.** Association between SDH level and progressions from prediabetes in transition pattern A using the multistate model, by adjusting for the overall lifestyle score in Model 2.

**Supplemental Figure 1.** Flowing chart of selecting participants.

**Supplemental Figure 2.** Numbers (percentages) of participants in transition from prediabetes to specific FCRMD, then to specific two CRMM, and ultimately to three CRMM.

**Supplemental Figure 3.** The transition probabilities in transition pattern A of unfavorable and favorable SDHs groups by using multistate model.

**Supplemental Figure 4.** Association between SDH level and progressions from prediabetes in the transition from prediabetes to specific FCRMD, then to specific two CRMM, and ultimately to three CRMM, using the multistate model.

**Supplemental Methods**

We tested the rationale of assuming lifestyle as potential mediators of the association between SDHs and progressions of prediabetes ^1,2^.

1. **Temporal precedence**

In our study, both SDHs (exposure) and lifestyle (mediator) were measured at baseline of the UK Biobank. This limited our ability to assess their temporal relationship. However, based on biological and social plausibility that SDHs typically precede and shape long-term lifestyle patterns ^3^, and in consistent with previous cohort studies which treated lifestyle as the mediator of the association between SDHs and health outcomes ^4,5^, we assume that theoretically, it is plausible to treat lifestyle as the mediator.

1. **Significant association between exposure and mediator**

We performed multinomial logistic regression to evaluate the association between SDH level (exposure) and lifestyle level (mediator), after adjusting for covariates. We yielded significant associations across different comparisons, indicating that those who had less favorable SDHs were more likely have less favorable lifestyle. For example, compared with people who had favorable SDHs, those had unfavorable SDHs was significantly associated with increased odds of having unfavorable lifestyle rather than favorable lifestyle (OR = 2.60, 95% CI :2.42, 2.79, *P* < 0.001).

| **SDH level** | **Lifestyle level** | **OR (95% CI)** | ***P*** |
| --- | --- | --- | --- |
| Favorable (Reference) |  |  |  |
| Medium | Medium vs Favorable | 1.17 (1.10, 1.25) | < 0.001 |
| Medium | Unfavorable vs Favorable | 1.48 (1.38, 1.59) | < 0.001 |
| Unfavorable | Medium vs Favorable | 1.57 (1.47, 1.67) | < 0.001 |
| Unfavorable | Unfavorable vs Favorable | 2.60 (2.42, 2.79) | < 0.001 |

1. **Significant association between mediator and outcome**

We used the multistate model to evaluate the association between lifestyle level (mediator) and progressions of prediabetes (outcomes), after adjusting for SDH level and other covariates. We found that the unfavorable lifestyle level was significantly associated with elevated risks for different transition stages, except for CRMM-to-death transition.

| **Lifestyle level** | **HR (95% CI)** | ***P*** |
| --- | --- | --- |
| **Prediabetes to FCRMD** |  |  |
| Favorable | Reference | / |
| Medium | 1.12 (1.06, 1.18) | < 0.001 |
| Unfavorable | 1.30 (1.24, 1.37) | < 0.001 |
| **Prediabetes to death** |  |  |
| Favorable | Reference | / |
| Medium | 1.31 (1.12, 1.53) | < 0.001 |
| Unfavorable | 1.74 (1.50, 2.03) | < 0.001 |
| **FCRMD to CRMM** |  |  |
| Favorable | Reference | / |
| Medium | 1.10 (0.98, 1.24) | 0.102 |
| Unfavorable | 1.20 (1.08, 1.35) | 0.001 |
| **FCRMD to death** |  |  |
| Favorable | Reference | / |
| Medium | 1.13 (0.95, 1.35) | 0.163 |
| Unfavorable | 1.42 (1.20, 1.68) | < 0.001 |
| **CRMM to death** |  |  |
| Favorable | Reference | / |
| Medium | 1.05 (0.79, 1.39) | 0.742 |
| Unfavorable | 1.27 (0.98, 1.65) | 0.073 |

1. **No confounding**

Our mediation analysis assumes no unmeasured confounding for (a) the exposure-outcome association, (b) the exposure-mediator association, and (c) the mediator-outcome association. Although we have adjusted for potential confounders and performed sensitivity analyses by further adjusting for medication use or cardiovascular and renal biomarkers, potential unmeasured confounding may not be ruled out due to the observational nature. We have acknowledged this in the limitation part.

**References:**

1. Baron RM, Kenny DA. The moderator-mediator variable distinction in social psychological research: conceptual, strategic, and statistical considerations. *Journal of personality and social psychology.* 1986;51(6):1173-1182. <https://doi.org/10.1037//0022-3514.51.6.1173>.

2. MacKinnon DP, Lockwood CM, Hoffman JM, West SG, Sheets V. A comparison of methods to test mediation and other intervening variable effects. *Psychological methods.* 2002;7(1):83-104. <https://doi.org/10.1037/1082-989x.7.1.83>.

3. Hill-Briggs F, Adler NE, Berkowitz SA, et al. Social Determinants of Health and Diabetes: A Scientific Review. *Diabetes Care.* 2020;44(1):258-279. <https://doi.org/10.2337/dci20-0053>.

4. Zhang YB, Chen C, Pan XF, et al. Associations of healthy lifestyle and socioeconomic status with mortality and incident cardiovascular disease: two prospective cohort studies. *Bmj.* 2021;373:n604. <https://doi.org/10.1136/bmj.n604>.

5. Petrovic D, de Mestral C, Bochud M, et al. The contribution of health behaviors to socioeconomic inequalities in health: A systematic review. *Preventive medicine.* 2018;113:15-31. <https://doi.org/10.1016/j.ypmed.2018.05.003>.

**Supplemental Table 1.** **Missing numbers and percentages of each SDHs and lifestyle component**

| **Variables** | **Missing number (percentage)** |
| --- | --- |
| **SDHs** |  |
| **Economic stability** |  |
| Household income | 8,902 (17%) |
| Employment status | 696 (1.4%) |
| Income quality | 1,306 (2.6%) |
| **Education access and quality** |  |
| Educational attainment | 793 (1.6%) |
| Education quality | 1,306 (2.6%) |
| **Health care access and quality** |  |
| Healthcare | 1,306 (2.6%) |
| **Neighborhood and built environment** |  |
| Accommodation stability | 668 (1.3%) |
| Housing quality | 1,306 (2.6%) |
| Local crime | 1,524 (3.0%) |
| Natural environment | 426 (0.8%) |
| **Social and community context** |  |
| Psychosocial problems | 624 (1.2%) |
| Race | 343 (0.7%) |
| Living alone | 580 (1.1%) |
| Social support | 2,470 (4.9%) |
| Social activity | 367 (0.7%) |
| Social isolation | 517 (1.0%) |
| Emotional distress | 620 (1.2%) |
| **Lifestyle** |  |
| Healthy diet | 136 (0.3%) |
| Never smoking | 330 (0.6%) |
| No excessive drinking | 182 (0.4%) |
| Healthy sleep | 86 (0.2%) |
| Healthy physical activity | 1,682 (3.3%) |

The percentage was calculated as the number of missing values divided by the number of participants with prediabetes and free of any cardio-renal-metabolic disease (CRMD) at baseline (n = 50,914).

SDHs: social determinants of health.

**Supplement Table 2. Comparison of characteristics between participants who were excluded due to missing data on any component of SDHs or lifestyle versus who were analyzed**

| **Characteristics** | **Participants who were excluded due to missing data (n = 13,816)** | **Participants who were analyzed (n = 37,098)** | ***P*** |
| --- | --- | --- | --- |
| **Age (years)** | 59.6 ± 7.2 | 58.8 ± 7.1 | < 0.001 |
| **Female (%)** | 8,680 (62.8) | 20,067 (54.1) | < 0.001 |
| **BMI (kg/m^2^)** | 28.8 ± 5.2 | 28.8 ± 5.3 | 0.811 |
| **Healthy BMI (%)** | 3,112 (22.5) | 8,429 (22.7) | 0.638 |
| **Hypertension (%)** | 4,350 (31.5) | 11,870 (32.0) | 0.271 |
| **HbA_1c_ (mmol/mmol)** | 41.2 ± 2.1 | 41.1 ± 2.0 | < 0.001 |

Among 50,914 participants with prediabetes and free of any cardio-renal-metabolic disease (CRMD) at baseline, 13,816 were excluded due to missing data on any components of SDHs and/or lifestyle. 37,098 participants were finally included in the current analysis.

Healthy BMI was defined as a BMI ≥ 18.5 kg/m^2^ and < 25 kg/m^2^.

SDHs: social determinants of health; BMI: body mass index.

**Supplemental Table 3. Assessment of SDHs in the UK Biobank**

| **Domain** | **Items** | **Definition (1 = advantaged level, 0 = disadvantaged level)** | **UK Biobank data field** |
| --- | --- | --- | --- |
| Economic stability | Household income | 0: Average total household income before tax ≥ £31,000  1: Lower than £31,000 | 738 |
|  | Employment status | 0: Employed, retired or student;  1: Unemployed | 6142 |
|  | Income quality | 0: Below the median of income deprivation score;  1: ≥ the median | 26411, 26418, 26428 |
| Education access and quality | Educational attainment | 0: Highest education level is college or above;  1: Lower than college | 6138 |
|  | Education quality | 0: Below the median of education deprivation score  1: ≥ the median | 26414, 26431, 26421 |
| Health care access and quality | Healthcare | 0: Below the median of healthcare deprivation score;  1: ≥ the median | 26413, 26430, 26420 |
| Neighborhood and built environment | Accommodation stability | 0: Own home outright;  1: Rent or other arrangement | 680 |
|  | Housing quality | 0: Below the median of housing deprivation score; 1: ≥ the median | 26415, 26432, 26423 |
|  | Local crime | 0: Below the median of crime score;  1: ≥ the median | 26416, 26434, 26425 |
|  | Natural environment | 0: ≥ the median of percentage of home location buffer classed as natural land;  1: below the median | 24502 |
| Social and community context | Psychosocial problems | 1: With diagnosed psychosocial problems including anxiety, depression and other mood disorders;  0: None | 2100, 2090, 130894, 130898, 130896, 130900, 130902, 130904, 130906 |
|  | Race | 1: Black Race;  0: Others | 21000 |
|  | Living alone | 0: Living with partners;  1: Living alone | 709 |
|  | Social support | 0: Able to confide in anyone close to you more often than once a week;  1 = once a week or less often | 2110 |
|  | Social activity | 0: Attend any group activities once a week or more often;  1: Less often than once a week | 6160 |
|  | Social isolation | 0: Visit friend/family or have them visit you more often than once a  week;  1: Once a week or less often | 1031 |
|  | Emotional distress | 0: None  1: Have experienced illness, injury, bereavement, stress within last 2 years | 6145 |

SDHs: social determinants of health.

**Supplemental Table 4. International Classification of Disease and procedure codes used to ascertain incident outcomes**

| **Outcomes** | **ICD-10 and/or OPCS-4 codes** |
| --- | --- |
| CVD | ICD-10: I20, I21, I22, I23, I24, I25, I48, I50, I60, I61, I62, I63, I64, I70.0, I70.2, I70.8, I70.9, I73.8, I73.9  OPCS-4: X09.3, X09.4, X09.5, L21.6, L51.3, L51.6, L51.8, L52.1, L52.2, L54.1, L54.4, L54.8, L59.1, L59.2, L59.3, L59.4, L59.5, L59.6, L59.7, L59.8, L60.1, L60.2, L63.1, L63.5, L63.9, L66.7 |
| T2D | E11 |
| CKD | N18 |

ICD-10: International Classification of Diseases 10th revision; OPCS-4: Office of Population Censuses and Surveys Classification of Interventions and Procedures, version 4; CVD: cardiovascular disease; T2D: type 2 diabetes; CKD: chronic kidney disease.

CVD included coronary heart disease, stroke, heart failure, atrial fibrillation and peripheral artery disease.

**Supplemental Table 5. Proportions of participants with disadvantaged levels for each SDHs item**

| **SDHs items** | **Favorable SDHs (n = 11,786)** | **Medium SDHs (n = 13,567)** | **Unfavorable SDHs (n = 11,745)** | **Overall (n = 37,098)** |
| --- | --- | --- | --- | --- |
| Household income | 4,209 (20.2%) | 7,489 (36.0%) | 9,096 (43.7%) | 20,794 |
| Employment status | 193 (8.4%) | 507 (22.2%) | 1,587 (69.4%) | 2,287 |
| Income quality | 1,372 (6.8%) | 7,779 (38.5%) | 11,051 (54.7%) | 20,202 |
| Educational attainment | 6,296 (24.4%) | 9,680 (37.5%) | 9,813 (38.1%) | 25,789 |
| Education quality | 1,514 (8.2%) | 6,946 (37.4%) | 10,099 (54.4%) | 18,559 |
| Healthcare | 1,742 (9.4%) | 6,877 (37.0%) | 9,979 (53.7%) | 18,598 |
| Accommodation stability | 2,972 (18.6%) | 5,346 (33.4%) | 7,667 (48.0%) | 15,985 |
| Housing quality | 4,595 (24.7%) | 6,531 (35.2%) | 7,445 (40.1%) | 18,571 |
| Local crime | 1,989 (10.7%) | 6,755 (36.3%) | 9,876 (53.0%) | 18,620 |
| Natural environment | 3,353 (18.1%) | 6,634 (35.8%) | 8,555 (46.1%) | 18,542 |
| Psychosocial problems | 2,710 (20.3%) | 4,765 (35.7%) | 5,877 (44.0%) | 13,352 |
| Race | 21 (2.1%) | 148 (14.7%) | 838 (83.2%) | 1,007 |
| Living alone | 996 (12.3%) | 2,556 (31.5%) | 4,565 (56.2%) | 8,117 |
| Social support | 2,778 (19.3%) | 5,080 (35.3%) | 6,528 (45.4%) | 14,386 |
| Social activity | 2,159 (18.6%) | 4,212 (36.4%) | 5,212 (45.0%) | 11,583 |
| Social isolation | 5,511 (26.9%) | 7,390 (36.1%) | 7,551 (36.9%) | 20,452 |
| Emotional distress | 3,353 (20.2%) | 5,934 (35.7%) | 7,341 (44.1%) | 16,628 |

Data are presented as n (row %).

SDHs: social determinants of health.

**Supplemental Table 6. Associations of SDH levels with risks of FCRMD, CRMM, and death estimated by the Cox model**

| **SDH level** | **FCRMD** | |  | **CRMM** | |  | **Death** | |
| --- | --- | --- | --- | --- | --- | --- | --- | --- |
|  | **Model 1** | **Model 2** |  | **Model 1** | **Model 2** |  | **Model 1** | **Model 2** |
| Favorable | Reference | / |  | Reference | / |  | Reference | / |
| Medium | 1.14 (1.09, 1.19) | 1.12 (1.07, 1.17) |  | 1.22 (1.10, 1.35) | 1.19 (1.08, 1.32) |  | 1.20 (1.10, 1.32) | 1.16 (1.06, 1.27) |
| Unfavorable | 1.36 (1.30, 1.43) | 1.32 (1.26, 1.38) |  | 1.61 (1.46, 1.78) | 1.55 (1.40, 1.72) |  | 1.68 (1.54, 1.84) | 1.57 (1.44, 1.72) |

Model 1 was adjusted for age, sex, healthy BMI status, prevalence of hypertension, and HbA_1c_. Model 2 was further adjusted for levels of overall lifestyle score.

Data are presented as HR (95% CI). All *P* < 0.001.

SDHs: social determinants of health; FCRMD: first cardio-renal-metabolic disease; CRMM: cardio-renal-metabolic multimorbidity.

**Supplemental Table 7.** **Association between SDH level and progressions from prediabetes in the transition pattern A, after** **excluded participants (n = 1707) occurred the outcomes in the first two-years of follow-up**

| **SDH level** | **No of events** | **Model 1** | |  | **Model 2** | |
| --- | --- | --- | --- | --- | --- | --- |
|  |  | **HR (95% CI)** | ***P*** |  | **HR (95% CI)** | ***P*** |
| **Prediabetes to FCRMD** |  |  |  |  |  |  |
| Favorable | 2759 | Reference | / |  | Reference | / |
| Medium | 3584 | 1.15 (1.09, 1.21) | <0.001 |  | 1.13 (1.07, 1.19) | <0.001 |
| Unfavorable | 3549 | 1.38 (1.31, 1.45) | <0.001 |  | 1.34 (1.27, 1.41) | <0.001 |
| **Prediabetes to death** |  |  |  |  |  |  |
| Favorable | 386 | Reference | / |  | Reference | / |
| Medium | 440 | 1.11 (0.96, 1.27) | 0.149 |  | 1.07 (0.93, 1.23) | 0.340 |
| Unfavorable | 423 | 1.45 (1.26, 1.67) | <0.001 |  | 1.35 (1.17, 1.55) | <0.001 |
| **FCRMD to CRMM** |  |  |  |  |  |  |
| Favorable | 554 | Reference | / |  | Reference | / |
| Medium | 780 | 1.12 (1.00, 1.25) | 0.045 |  | 1.10 (0.99, 1.23) | 0.078 |
| Unfavorable | 826 | 1.32 (1.19, 1.48) | <0.001 |  | 1.30 (1.16, 1.45) | <0.001 |
| **FCRMD to death** |  |  |  |  |  |  |
| Favorable | 269 | Reference | / |  | Reference | / |
| Medium | 336 | 1.10 (0.94, 1.29) | 0.237 |  | 1.08 (0.92, 1.26) | 0.374 |
| Unfavorable | 409 | 1.60 (1.37, 1.88) | <0.001 |  | 1.53 (1.31, 1.79) | <0.001 |
| **CRMM to death** |  |  |  |  |  |  |
| Favorable | 92 | Reference | / |  | Reference | / |
| Medium | 156 | 1.30 (1.00, 1.68) | 0.048 |  | 1.26 (0.97, 1.64) | 0.079 |
| Unfavorable | 157 | 1.30 (1.00, 1.68) | 0.051 |  | 1.24 (0.95, 1.62) | 0.106 |

Model 1 was adjusted for age, sex, healthy BMI status, prevalence of hypertension, and HbA_1c_. Model 2 was further adjusted for levels of overall lifestyle score.

SDHs: social determinants of health; FCRMD: first cardio-renal-metabolic disease; CRMM: cardio-renal-metabolic multimorbidity.

**Supplemental Table 8. Association between SDH level and progressions from prediabetes in the transition pattern A using multistate model further adjusting for drug use**

| **SDH level** | **No of events** | **HR (95% CI)** | ***P*** |
| --- | --- | --- | --- |
| **Prediabetes to FCRMD** |  |  |  |
| Favorable | 3198 | Reference | / |
| Medium | 4143 | 1.12 (1.07, 1.17) | < 0.001 |
| Unfavorable | 4105 | 1.32 (1.26, 1.38) | < 0.001 |
| **Prediabetes to death** |  |  |  |
| Favorable | 419 | Reference | / |
| Medium | 504 | 1.13 (0.99, 1.28) | 0.073 |
| Unfavorable | 479 | 1.40 (1.22, 1.60) | < 0.001 |
| **FCRMD to CRMM** |  |  |  |
| Favorable | 664 | Reference | / |
| Medium | 934 | 1.12 (1.01, 1.24) | 0.028 |
| Unfavorable | 1011 | 1.35 (1.22, 1.50) | < 0.001 |
| **FCRMD to death** |  |  |  |
| Favorable | 316 | Reference | / |
| Medium | 407 | 1.11 (0.95, 1.28) | 0.184 |
| Unfavorable | 489 | 1.57 (1.35, 1.81) | < 0.001 |
| **CRMM to death** |  |  |  |
| Favorable | 119 | Reference | / |
| Medium | 184 | 1.14 (0.91, 1.44) | 0.256 |
| Unfavorable | 193 | 1.17 (0.92, 1.48) | 0.198 |

Model was adjusted for age, sex, healthy BMI status, prevalence of hypertension, and HbA_1c_, levels of overall lifestyle score, use of antihypertensive drugs use of statin.

SDHs: social determinants of health; FCRMD: first cardio-renal-metabolic disease; CRMM: cardio-renal-metabolic multimorbidity.

**Supplemental Table 9. Association between SDH level and progressions from prediabetes in the transition pattern A using multistate model further adjusting for serum LDL-C and eGFR**

| **SDH level** | **No of events** | **HR (95% CI)** | ***P*** |
| --- | --- | --- | --- |
| **Prediabetes to FCRMD** |  |  |  |
| Favorable | 3054 | Reference | / |
| Medium | 3940 | 1.12 (1.07, 1.17) | < 0.001 |
| Unfavorable | 3891 | 1.32 (1.25, 1.38) | < 0.001 |
| **Prediabetes to death** |  |  |  |
| Favorable | 399 | Reference | / |
| Medium | 473 | 1.11 (0.97, 1.27) | 0.136 |
| Unfavorable | 448 | 1.37 (1.20, 1.58) | < 0.001 |
| **FCRMD to CRMM** |  |  |  |
| Favorable | 634 | Reference | / |
| Medium | 886 | 1.12 (1.01, 1.24) | 0.036 |
| Unfavorable | 977 | 1.38 (1.25, 1.53) | < 0.001 |
| **FCRMD to death** |  |  |  |
| Favorable | 304 | Reference | / |
| Medium | 391 | 1.10 (0.94, 1.27) | 0.234 |
| Unfavorable | 465 | 1.55 (1.33, 1.80) | < 0.001 |
| **CRMM to death** |  |  |  |
| Favorable | 112 | Reference | / |
| Medium | 175 | 1.17 (0.92, 1.49) | 0.197 |
| Unfavorable | 188 | 1.19 (0.93, 1.51) | 0.158 |

Model was adjusted for age, sex, healthy BMI status, prevalence of hypertension, and HbA_1c_, levels of overall lifestyle score, serum LDL-C and eGFR.

This analysis was conducted among 35,269 participants who had complete data on serum LDL-C and eGFR.

SDHs: social determinants of health; FCRMD: first cardio-renal-metabolic disease; CRMM: cardio-renal-metabolic multimorbidity. LDL-C: low-density lipoprotein cholesterol; eGFR: estimated glomerular filtration rate.

**Supplemental Table 10. Association between SDH level and progressions from prediabetes in the transition pattern A, using different intervals for the participants entering different states on the same day**

| **SDH level** | **1 day interval** | |  | **30-days interval** | |  | **365-days interval** | |
| --- | --- | --- | --- | --- | --- | --- | --- | --- |
|  | **HR (95% CI)** | ***P*** |  | **HR (95% CI)** | ***P*** |  | **HR (95% CI)** | ***P*** |
| **Prediabetes to FCRMD** |  |  |  |  |  |  |  |  |
| Favorable | Reference | / |  | Reference | / |  | Reference | / |
| Medium | 1.12 (1.07, 1.17) | <0.001 |  | 1.12 (1.07, 1.17) | <0.001 |  | 1.12 (1.07, 1.17) | <0.001 |
| Unfavorable | 1.32 (1.26, 1.38) | <0.001 |  | 1.32 (1.26, 1.38) | <0.001 |  | 1.31 (1.25, 1.38) | <0.001 |
| **Prediabetes to death** |  |  |  |  |  |  |  |  |
| Favorable | Reference | / |  | Reference | / |  | Reference | / |
| Medium | 1.12 (0.99, 1.28) | 0.078 |  | 1.12 (0.98, 1.28) | 0.084 |  | 1.12 (0.98, 1.27) | 0.094 |
| Unfavorable | 1.39 (1.22, 1.59) | <0.001 |  | 1.40 (1.22, 1.60) | <0.001 |  | 1.42 (1.24, 1.62) | <0.001 |
| **FCRMD to CRMM** |  |  |  |  |  |  |  |  |
| Favorable | Reference | / |  | Reference | / |  | Reference | / |
| Medium | 1.12 (1.01, 1.23) | 0.031 |  | 1.12 (1.01, 1.24) | 0.029 |  | 1.12 (1.01, 1.23) | 0.032 |
| Unfavorable | 1.35 (1.22, 1.49) | <0.001 |  | 1.35 (1.22, 1.49) | <0.001 |  | 1.33 (1.20, 1.47) | <0.001 |
| **FCRMD to death** |  |  |  |  |  |  |  |  |
| Favorable | Reference | / |  | Reference | / |  | Reference | / |
| Medium | 1.10 (0.95, 1.27) | 0.225 |  | 1.10 (0.94, 1.27) | 0.228 |  | 1.10 (0.95, 1.27) | 0.211 |
| Unfavorable | 1.56 (1.35, 1.81) | <0.001 |  | 1.56 (1.34, 1.80) | <0.001 |  | 1.55 (1.34, 1.79) | <0.001 |
| **CRMM to death** |  |  |  |  |  |  |  |  |
| Favorable | Reference | / |  | Reference | / |  | Reference | / |
| Medium | 1.15 (0.91, 1.45) | 0.240 |  | 1.15 (0.91, 1.45) | 0.234 |  | 1.16 (0.92, 1.46) | 0.223 |
| Unfavorable | 1.16 (0.92, 1.47) | 0.210 |  | 1.16 (0.92, 1.47) | 0.207 |  | 1.16 (0.91, 1.47) | 0.221 |

Model was adjusted for age, sex, healthy BMI status, prevalence of hypertension, and HbA_1c_, levels of overall lifestyle score.

SDHs: social determinants of health; FCRMD: first cardio-renal-metabolic disease; CRMM: cardio-renal-metabolic multimorbidity.

**Supplemental Table 11. Association between SDH level and progressions from prediabetes in the transition pattern A, stratified analyses by sex and age group**

| **Transitions** | **HR (95% CI)** | | ***P* for interaction** |
| --- | --- | --- | --- |
| **Sex** | **Female** | **Male** |  |
| Prediabetes to FCRMD | 1.51 (1.40, 1.62) | 1.18 (1.11, 1.26) | < 0.001 |
| Prediabetes to death | 1.30 (1.08, 1.56) | 1.50 (1.24, 1.82) | 0.328 |
| FCRMD to CRMM | 1.27 (1.08, 1.50) | 1.38 (1.22, 1.57) | 0.462 |
| FCRMD to death | 1.42 (1.11, 1.82) | 1.65 (1.38, 1.98) | 0.469 |
| CRMM to death | 1.44 (0.94, 2.19) | 1.04 (0.78, 1.38) | 0.205 |
| **Age group** | **< 60 years old** | **≥ 60 years old** |  |
| Prediabetes to FCRMD | 1.35 (1.24, 1.46) | 1.25 (1.18, 1.33) | 0.020 |
| Prediabetes to death | 1.05 (0.83, 1.33) | 1.44 (1.23, 1.70) | 0.073 |
| FCRMD to CRMM | 1.51 (1.23, 1.85) | 1.29 (1.15, 1.45) | 0.143 |
| FCRMD to death | 1.38 (1.03, 1.84) | 1.54 (1.30, 1.83) | 0.451 |
| CRMM to death | 1.49 (0.77, 2.88) | 1.09 (0.85, 1.41) | 0.425 |

HR (95% CI) comparing those with unfavorable SDH level versus those with favorable SDH level are shown.

Model was adjusted for age, sex, healthy BMI status, prevalence of hypertension, and HbA_1c_, levels of overall lifestyle score.

SDHs: social determinants of health; FCRMD: first cardio-renal-metabolic disease; CRMM: cardio-renal-metabolic multimorbidity.

**Supplemental Table 12. Association between SDH level and progressions from prediabetes in transition pattern A using the multistate model, by adjusting for the overall lifestyle score in Model 2**

| **SDH level** | **No of events** | **Model 1** | |  | **Model 2** | | **Mediation proportion (%) (95% CI)** |
| --- | --- | --- | --- | --- | --- | --- | --- |
|  |  | **HR (95% CI)** | ***P*** |  | **HR (95% CI)** | ***P*** |  |
| **Prediabetes to FCRMD** |  |  |  |  |  |  |  |
| Favorable | 3198 | Reference | / |  | Reference | / |  |
| Medium | 4143 | 1.14 (1.09, 1.19) | < 0.001 |  | 1.12 (1.07, 1.17) | < 0.001 | 21.1 (13.1, 32.2) |
| Unfavorable | 4105 | 1.36 (1.30, 1.43) | < 0.001 |  | 1.31 (1.25, 1.37) | < 0.001 | 16.3 (13.0, 20.3) |
| **Prediabetes to death** |  |  |  |  |  |  |  |
| Favorable | 419 | Reference | / |  | Reference | / |  |
| Medium | 504 | 1.16 (1.02, 1.33) | 0.022 |  | 1.11 (0.98, 1.27) | 0.104 | 31.1 (10.0, 64.6) |
| Unfavorable | 479 | 1.50 (1.31, 1.71) | < 0.001 |  | 1.37 (1.20, 1.57) | < 0.001 | 23.4 (15.2, 34.3) |
| **FCRMD to CRMM** |  |  |  |  |  |  |  |
| Favorable | 664 | Reference | / |  | Reference | / |  |
| Medium | 934 | 1.13 (1.02, 1.25) | 0.017 |  | 1.11 (1.01, 1.23) | 0.039 | 18.5 (5.1, 49.0) |
| Unfavorable | 1011 | 1.38 (1.24, 1.52) | < 0.001 |  | 1.33 (1.21, 1.48) | < 0.001 | 12.0 (6.8, 20.3) |
| **FCRMD to death** |  |  |  |  |  |  |  |
| Favorable | 316 | Reference | / |  | Reference | / |  |
| Medium | 407 | 1.12 (0.97, 1.30) | 0.118 |  | 1.09 (0.94, 1.26) | 0.266 | / |
| Unfavorable | 489 | 1.65 (1.43, 1.90) | < 0.001 |  | 1.54 (1.33, 1.78) | < 0.001 | 17.1 (10.5, 26.5) |
| **CRMM to death** |  |  |  |  |  |  |  |
| Favorable | 119 | Reference | / |  | Reference | / |  |
| Medium | 184 | 1.18 (0.93, 1.48) | 0.170 |  | 1.15 (0.91, 1.46) | 0.227 | / |
| Unfavorable | 193 | 1.21 (0.96, 1.52) | 0.114 |  | 1.16 (0.92, 1.47) | 0.204 | / |

Model 1 was adjusted for age, sex, healthy BMI status, prevalence of hypertension, and HbA_1c_. Model 2 was further adjusted for overall lifestyle score. The mediation proportion of lifestyle was calculated when SDHs shown a significant association in the Model 1, by using the difference methods which comparing the estimates of SDHs between Model 1 and Model 2.

SDHs: social determinants of health; FCRMD: first cardio–renal–metabolic disease; CRMM: cardio–renal–metabolic multimorbidity.

**Supplemental Figure 1. Flowing chart of selecting participants**

CRMD: cardio-renal-metabolic disease; eGFR: estimated glomerular filtration rate; UACR: urinary albumin to urinary creatinine ratio; SDHs: social determinants of health

**
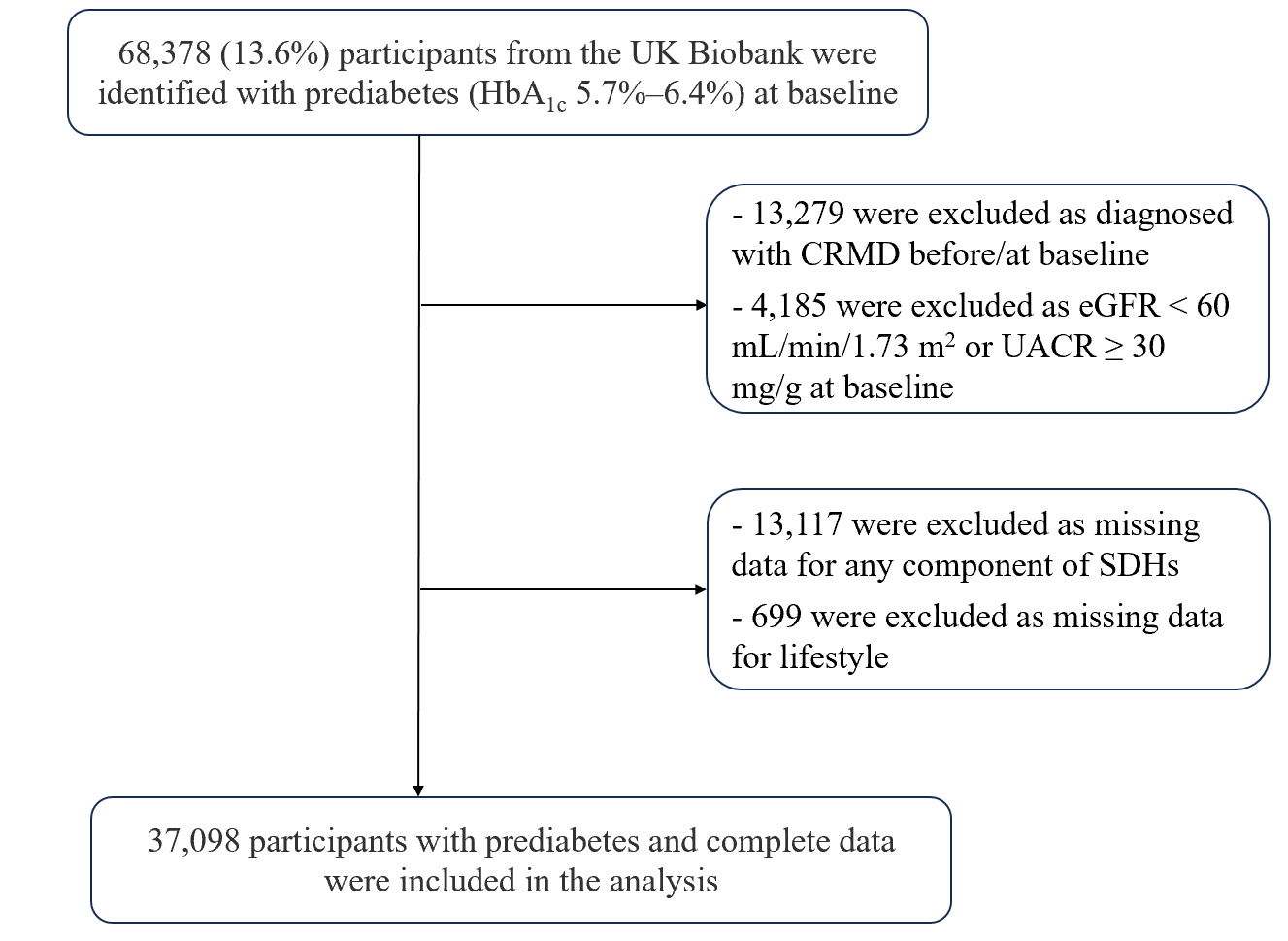
**

**Supplemental Figure 2. Numbers (percentages) of participants in transition from prediabetes to specific FCRMD, then to specific two CRMM, and ultimately to three CRMM**

CRMD: cardio-renal-metabolic disease; CRMM: cardio-renal-metabolic multimorbidity (the coexistence of two or three CRMDs); CVD: cardiovascular disease; T2D: type 2 diabetes, CKD: chronic kidney disease.

CRMDs included CVD, T2D, and CKD.


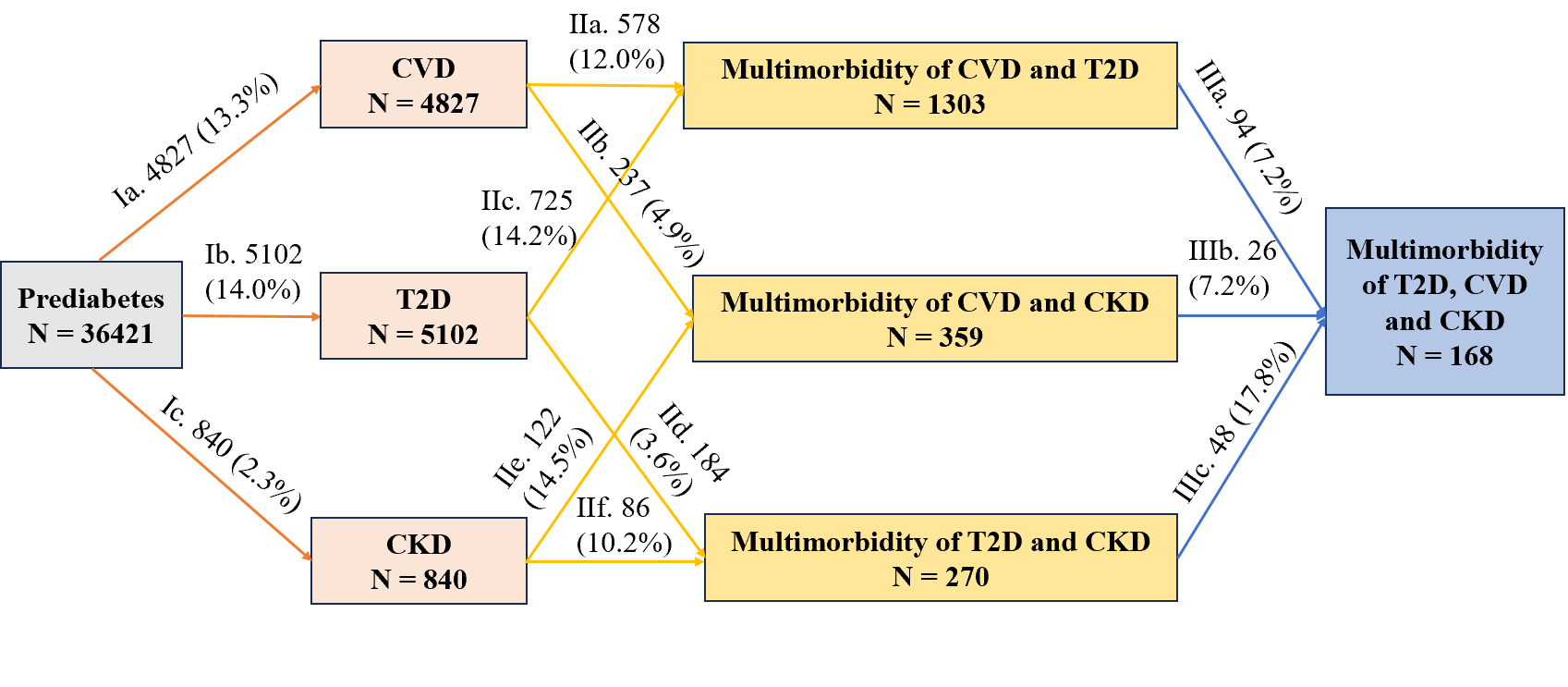


**Supplemental Figure 3. The transition probabilities in transition pattern A of unfavorable and favorable SDHs groups by using multistate model**

SDHs: social determinants of health; FCRMD: first cardio-renal-metabolic disease; CRMM: cardio-renal-metabolic multimorbidity.

The probabilities between two groups were compared using paired sample t-tests.

**
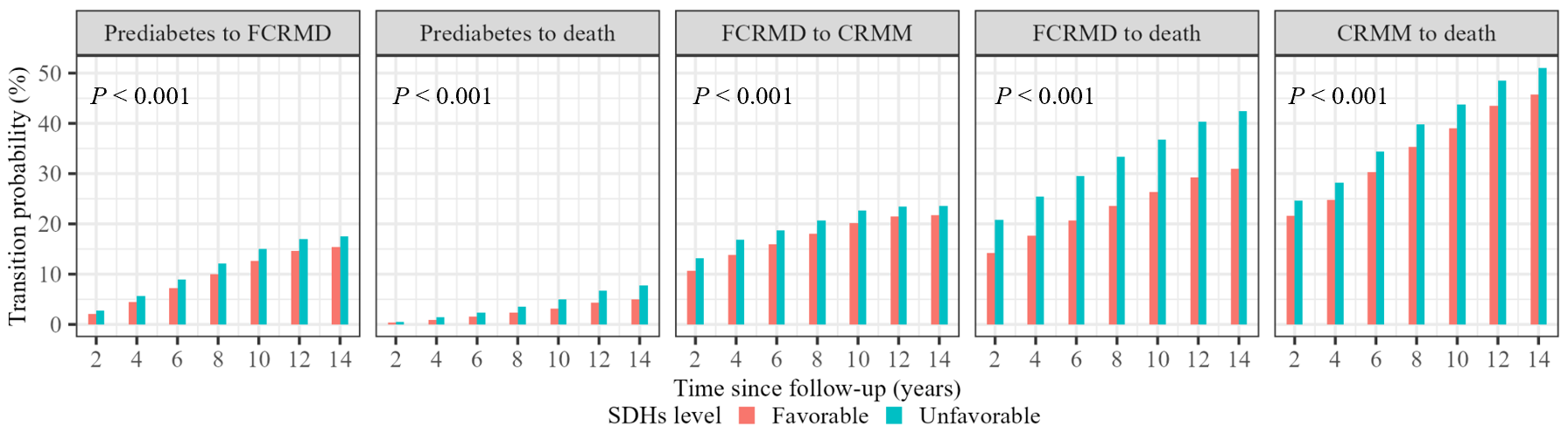
**

**Supplemental Figure 4. Association between SDH level and progressions from prediabetes in the transition from prediabetes to specific FCRMD, then to specific two CRMM, and ultimately to three CRMM, using the multistate model**

Model was adjusted for age, sex, healthy BMI status, prevalence of hypertension, and HbA_1c_, level of overall lifestyle score.

SDHs: social determinants of health; FCRMD: first cardio-renal-metabolic disease; CRMM: cardio-renal-metabolic multimorbidity. CVD: cardiovascular disease; T2D: type 2 diabetes, CKD: chronic kidney disease. CRMDs included CVD, T2D, and CKD.


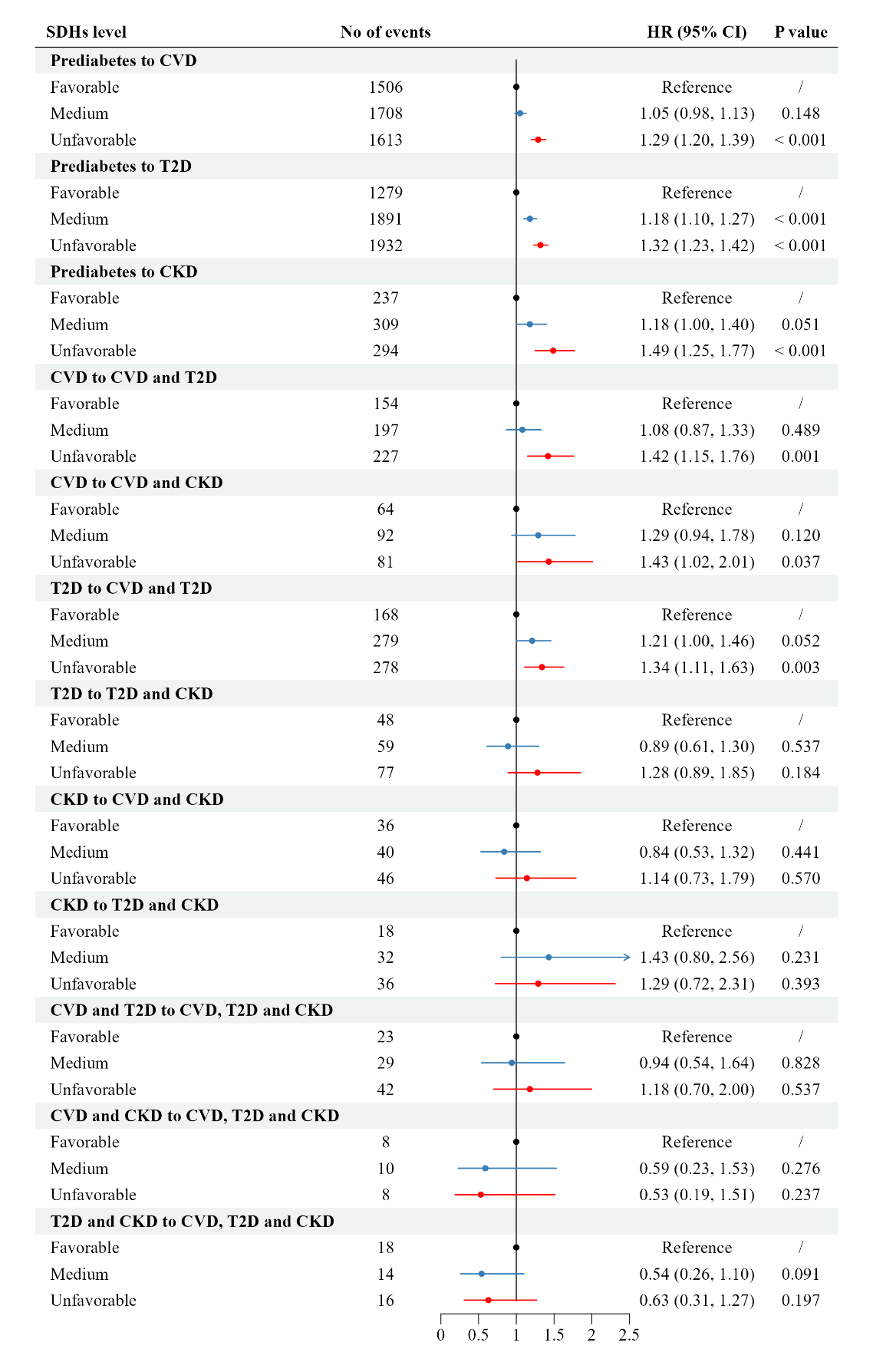

Supplement: Supplementary file 1 — Data S1. Supporting Information. [file DOM-27-6605-s001.docx]
